# A blood test-based machine learning model for predicting lung cancer risk

**DOI:** 10.3389/fmed.2025.1577451

**Published:** 2025-06-17

**Authors:** Lihi Schwartz, Naor Matania, Matanel Levi, Teddy Lazebnik, Shiri Kushnir, Noga Yosef, Assaf Hoogi, Dekel Shlomi

**Affiliations:** ^1^Fliner Clinic, Department of Family Medicine, Dan-Petah-Tiqwa District, Clalit Health Services Community Division, Petah Tiqwa, Israel; ^2^Department of Computer Science, Bar Ilan University, Ramat Gan, Israel; ^3^Adelson School of Medicine, Ariel University, Ariel, Israel; ^4^Department of Mathematics, Ariel University, Ariel, Israel; ^5^Department of Cancer Biology, Cancer Institute, University College London, London, United Kingdom; ^6^Research Authority, Rabin Medical Center, Beilinson Campus, Petah Tiqwa, Israel; ^7^Research Unit, Dan-Petah-Tiqwa District, Clalit Health Services Community Division, Ramat Gan, Israel; ^8^The School of Computer Science and The Data Science and Artificial Intelligence Research Center, Ariel University, Ariel, Israel; ^9^Pulmonary Clinic, Dan-Petah-Tiqwa District, Clalit Health Services Community Division, Ramat Gan, Israel

**Keywords:** lung cancer, artificial intelligence, machine learning, blood test, prediction model

## Abstract

**Background:**

The goal of early detection is individual cancer prediction. For lung cancer (LC), age and smoking history are the primary criteria for annual low-dose CT screening, leaving other populations at risk of being overlooked. Machine learning (ML) is a promising method to identify complex patterns in the data that can reveal personalized disease predictors.

**Methods:**

An ML-based model was used on blood test data collected before the diagnosis of LC, and sociodemographic factors such as age and gender among LC patients and controls were incorporated to predict the risk for future LC diagnosis.

**Results:**

In addition to age and gender, we identified 22 blood tests that contributed to the model. For the entire study population, the ML model predicted LC with an accuracy of 71.2%, a sensitivity of 63%, and a positive predictive value of 67.2%. Higher accuracy was found among women than men (71.8 vs. 70.8) and among never smokers than smokers (73.6 vs. 70.1%). Age was the most significant contributor (13.6%), followed by red blood cell distribution (5.1%), creatinine (5%), gender (3.6%), and mean corpuscular hemoglobin (3.3%). A majority of the blood tests made a highly variable contribution to the complex ML model; however, some tests, such as red cell distribution width, mean corpuscular hemoglobin, prothrombin time, hematocrit, urea, and calcium, contributed slightly more to a dichotomous prediction.

**Conclusion:**

Blood tests can be used in the proposed ML model to predict LC. More studies are needed in basic science fields to identify possible explanations between specific blood results and LC prediction.

## Introduction

According to the World Health Organization (WHO), lung cancer (LC) is the leading cause of cancer-related death worldwide, with 1.2 million global deaths a year (1). The main risk factors for LC are age and smoking (2). In general, the goal of screening tests is to reduce mortality through early detection and effective treatment, as symptoms typically manifest during advanced stages (2).

The U.S. Preventive Services Task Force (USPSTF) currently recommends annual chest low-dose computed tomography (LDCT) for individuals aged 50–80 years who have a smoking history of at least 20 pack-years and who are either current smokers or have quit within the last 15 years all three criteria must be met (3). Recently, the American Cancer Society (ACS) published updated recommendations for LC screening criteria using chest LDCT. The new recommendations eliminate the criterion of having quit smoking within the last 15 years (4).

Nevertheless, current LC screening recommendations outlined by leading organizations such as the National Comprehensive Cancer Network (NCCN) and U.S. Preventive Services Task Force (USPSTF) do not target specific populations with lower risk factors, such as never smokers, light smokers, and passive smokers. These special populations need specific recommendations, which are lacking. For example, a recent report by the ACS highlighted the concerning, unclear trend of a higher LC incidence among women aged 35 to 54 years than among men in the same age group—a reversal of the historically higher burden in men (5).

Furthermore, the rate of eligible individuals performing the screening program is reported to be low. For example, a systematic review and meta-analysis conducted in the United States found that annual screening using LDCT after the baseline scan is very low, ranging from 44 to 66% (6). Several recent studies have reported similar low adherence rates, ranging from 41 to 63% (7–9).

The use of tumor marker results, such as carcinoembryonic antigen (CEA), in the diagnosis of LC remains unreliable due to the markers' relatively low levels of specificity and sensitivity (10). To date, no blood test can serve as a marker for LC screening.

While the combination of additional laboratory indicators holds promise for improving the diagnostic efficiency of LC markers, the complex nature of the data poses a challenge in identification (11). As an example, in early-stage non-small cell lung cancer (NSCLC), the utility of neuron-specific enolase (NSE), CA125, and squamous cell carcinoma antigen (SCC) was found to be limited, even when these three markers were used in combination (12). In a systematic review, the value of “old” laboratory tests, such as calcium, albumin, or other proteins, and blood cell counts, was evaluated as prognostic factors in LC (13).

One way to address the limitations associated with LDCT screening is to utilize artificial intelligence (AI)-based models. For example, machine learning (ML), a branch of AI, can analyze large amounts of data in electronic medical records (EMRs) and identify complex patterns in them that serve as the basis for a wide range of clinical decisions (14, 15). Rule-based AI systems have demonstrated varying degrees of clinical efficacy in addressing various aspects of LC, including diagnosis, treatment, and prognosis (11). Incorporating AI into LC imaging analysis can enhance the precision of LC screening, reduce analysis time, and enhance the overall efficiency of clinicians (16). For instance, Lazebnik et al. developed a clinical model to forecast the spatial dissemination of LC metastasis, with an accuracy rate of 74% (17).

Since blood tests are intensively used in both inpatient and community settings, they may provide important information concerning cancer risk. Although a single blood test result may not predict cancer risk, the complex interactions between multiple tests can be explored by AI, which may shed light on this domain. Therefore, using blood tests in an AI model can potentially stratify patients' individual risk of LC. With the use of AI, the integration of tumor markers with inflammatory or metabolic markers has been found to be a reliable strategy for diagnosing LC (18). In a previous study, the combination of human epidermal growth factor 4 (HE4), apolipoprotein A2 (ApoA2), sarcosine (TTR), and vascular cell adhesion molecule-1 (sVCAM-1), in conjunction with CEA, had an area under the receiver operating characteristic curve (AUC) of 0.98, with a sensitivity of 93% and specificity of 92% (18).

Furthermore, recently, in a large study, an AI model based on the EMRs of subjects with a positive fecal occult blood test identified subjects with more than twice the risk of colorectal cancer compared to a fecal occult blood test alone (19).

Recently, the International Association for the Study of Lung Cancer (IASLC) published a roadmap for LC screening that emphasizes the need to identify individuals who have never smoked but are at high risk for developing LC (20). One of the priorities for the coming years, according to the IASLC, is to integrate AI and biomarkers to improve the prediction of malignancy in suspicious CT screen-detected lesions.

The objective of this study is to design an ML-driven decision-making model that utilizes blood tests in patients with high and low risk for LC to evaluate individual risk for LC. Our hypothesis is that using blood tests in an AI model could predict the individual risk of LC. In the future, personalized recommendations for chest LDCT screening using AI and blood tests could be developed. We have designed this article in accordance with the Transparent Reporting of a multivariable prediction or diagnosis model (TRIPOD) reporting checklist.

## Methods

This retrospective study is based on the EMRs of patients in 'Clalit Health Services' (CHS), the largest publicly funded Health Maintenance Organization (HMO) in Israel, which is responsible for ~50% of the Israeli population. The inclusion criteria were adults (men and women) aged 35 years and older who underwent chest CT scans at a medical center or a clinic connected to the picture archiving and communication system (PACS) of the CHS between 1 January 2005 and 31 December 2022. We excluded patients who were diagnosed with cancer other than LC before undergoing the CT scan.

The study group included patients with chest CT scans performed up to ~5.5 years before the LC diagnosis. Similarly, their blood tests were also collected during the same period. For each LC patient, the control group included two matched participants who were randomly matched by a computer program according to age (by year of birth), gender, and smoking status (never smokers vs. those with any type of smoking history) who were not diagnosed with LC. Information regarding smoking status was recorded by the primary physicians. The patients were interviewed by physicians at least once during the study period. The study population included patients who underwent chest CT scans up to ~5.5 years before a diagnosis of LC. Patients in the control group who were not alive in the year their matched patients were diagnosed with LC were excluded.

For this study, we extracted all blood test results from the EMRs. Then, we grouped together the same blood tests with different names/abbreviations and converted different results in different units to a uniform value. The specific blood test result of an LC patient was matched to a control patient based on gender and age at the time of the blood test.

The study was approved by the Institutional Review Board (IRB) of the Meir Medical Center, Kfar-Saba, Israel (approval no. 0079-21-COM1).

### Statistical analysis

Comparisons between the groups were made using *t*-tests for continuous variables and chi-squared tests for categorical variables. The statistical analysis was conducted using the Python programming language (version 3.7.5) (21).

### Artificial intelligence model

To develop and analyze the ability to predict LC from these data, we first divided the dataset into training and validation cohorts, with the former comprising 80% of the data and the latter comprising the remaining 20%. We ensured that the training and testing data were statistically significantly similar in terms of the sociodemographic variables using a two-sided *t*-test (*p* < 0.05), while also maintaining the proportion of case-to-control samples in both cohorts within a 3% difference.

Based on these data, we used an exclusive ML pipeline. For the feature selection part, we used the top-K method, taking into account the most frequent features, such that *k* = 24 was found for features present in at least 2% of the population (22). Notably, the exact value of the 2% threshold was obtained via a trial-and-error approach aiming to maximize the F1-score of the obtained model. To this end, we also attempted principal component analysis (PCA) with multiple dimensions (ranging from 2 to 40) (23), forward selection (24), and backward elimination (25), which resulted in significantly worse results. We did not identify outliers for this cohort. As the data were encoded for the 24 features, lack of tests was represented by a null value (not a zero value), since imputation presents challenges with the current data due to its extreme sparsity in several features, where even the majority of patients lack data (26). Next, for the ML model itself, we used the CatBoost algorithm with hyperparameter optimization obtained using the grid-search method, optimizing the model's accuracy on the training cohort. Moreover, we used the SAT-based post-pruning method (27) on the model to improve its generalization capabilities. We chose CatBoost, as identified through an extensive search using TPOT (an automated Machine Learning tool) (28). CatBoost is well-suited for this task due to its robustness in terms of performance with limited hyperparameter tuning (29, 30), which can be useful in the clinical domain prone to concept drift (31); CatBoost's ability to handle missing values (i.e., nulls) out-of-the-box (32) is critical due to the extremely sparse nature of the data.

To explore the obtained model's decision-making process, we evaluated the importance, using the information gain method (33), of each model's parameters to learn the clinical reasoning revealed by the model.

Moreover, we compared the performance of the obtained model with three baseline models: a decision tree (DT) (34), a histogram-based gradient boosting model (HGBT) (35), and an XGboost model (36).

## Results

Of the 4,094 patients, 18 patients were excluded due to a lack of smoking history. The final study population consisted of 4,076 patients, with 1,428 (35%) LC patients and 2,648 (65%) matched patients in the control group (Table 1). The LC group had a higher proportion of patients with a positive history of smoking (current or past) compared to the control group (74 vs. 68%, respectively, *p* < 0.0001). Age and gender were similar between the two groups.

**Table 1:** Characteristics of the study population.

	**Total (*n* = 4,076)**	**Lung cancer (*n* = 1,428)**	**Without lung cancer (*n* = 2,648)**	***p*-Value**
**Age**				
Mean (SD)	69.51 (10.5)	69.63 (10.5)	69.44 (10.5)	0.58
**Gender**				
Male (%)	2,475 (60.7%)	876 (61.3%)	1,599 (60.4%)	0.55
**Smoking status**				
Never (%)	1,212 (29.7%)	371 (26.0%)	841 (31.8%)	< 0.001
Past or current (%)	2,864 (70.3%)	1,057 (74.0%)	1,807 (68.2%)	

### Blood tests on an artificial model

The performance of the AI model is represented in Table 2. For the entire study population, the validation cohort exhibited an accuracy of 71.2%, a recall (sensitivity) of 63%, a precision (positive predictive value; PPV) of 67.2%, a specificity of 77.2%, an F1-score of 65.1%, and an AUC of 78.7%. Women in the validation cohort showed slightly better performance than men, with an accuracy of 71.8 vs. 70.8%, respectively, a sensitivity of 64.5 vs. 62.1%, a PPV of 68.7 vs. 66.3%, an F1-score of 66.5 vs. 64.1%, a specificity of 77.5 vs. 77.0%, and an AUC of 79.7 vs. 78.0%. Never smokers in the validation cohort showed better performance than smokers in that cohort, with an accuracy of 73.6 vs. 70.1%, a sensitivity of 65.3 vs. 62.2%, but a lower PPV of 64.8 vs. 68.4%, an F1-score of 66.3 vs. 65.1%, a specificity of 77.1 vs. 76.6%, and an AUC of 79.5 vs. 78.2%, respectively.

**Table 2:** Performance of machine learning models including gender and smoking status analysis.

	**Accuracy**	Recall^*^	**Precision^#^**	**F1-score**	**Specificity**	**AUC^x^**
All	71.2%	63.0%	67.2%	65.0%	77.2%	78.7%
**Gender**						
Females	71.8%	64.5%	62.1%	72.5%	77.5%	79.8%
Males	70.8%	68.7%	66.3%	64.1%	77.0%	78.0%
**Smoking status**						
Past or current	70.1%	62.2%	68.4%	65.1%	74.3%	79.1%
Never-smokers	73.6%	65.3%	64.8%	65.0%	70.8%	78.4%

^*^Sensitivity.

^#^Positive predictive value.

^x^Area under the receiver operating characteristic curve.

**Figure 1:**
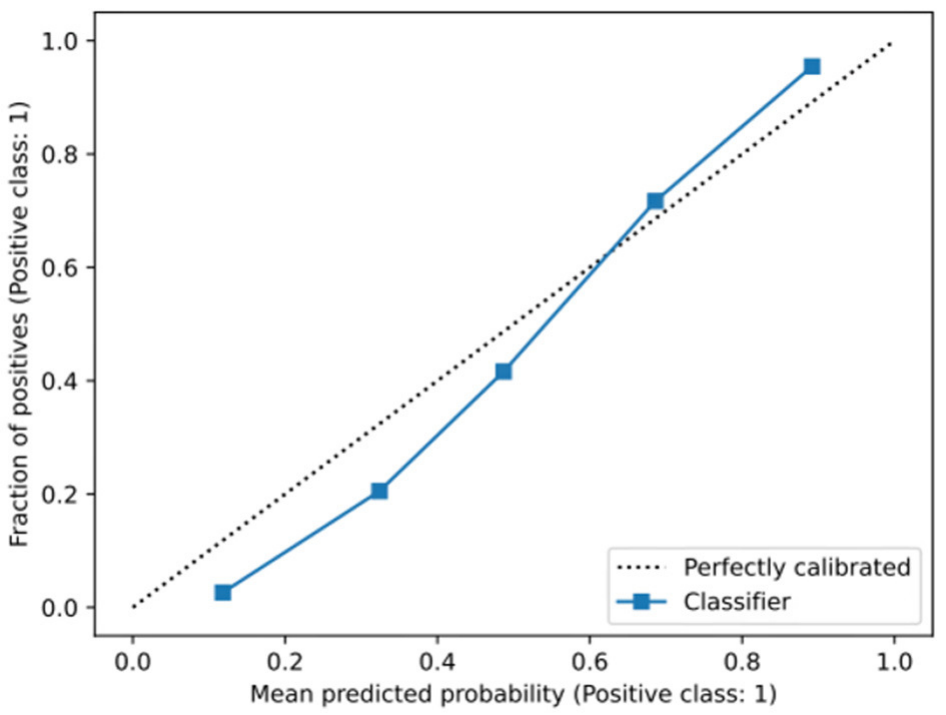
Area under the receiver operating characteristic curve of the obtained model.

Figure 1 shows the receiver operating characteristic (ROC) curve of the obtained model for the entire study population and the two subpopulations according to gender and smoking status. The 95% confidence interval (CI) of the obtained model's AUC was found to be between 78 and 79% with a Youden's *J* index of 0.41. Similarly, the 95% CIs of the obtained models' AUC for the gender-specific models were found to be 77%−79% and 79%−81% for men and women, respectively, with corresponding Youden's *J* indexes of 0.40 and 0.43. Smokers and non-smokers showed almost identical results, with 95% CIs of the obtained models' AUC being 77%−79% and 80%−82%, respectively, with corresponding Youden's *J* indexes of 0.39 and 0.45, respectively.

**Figure 2:**
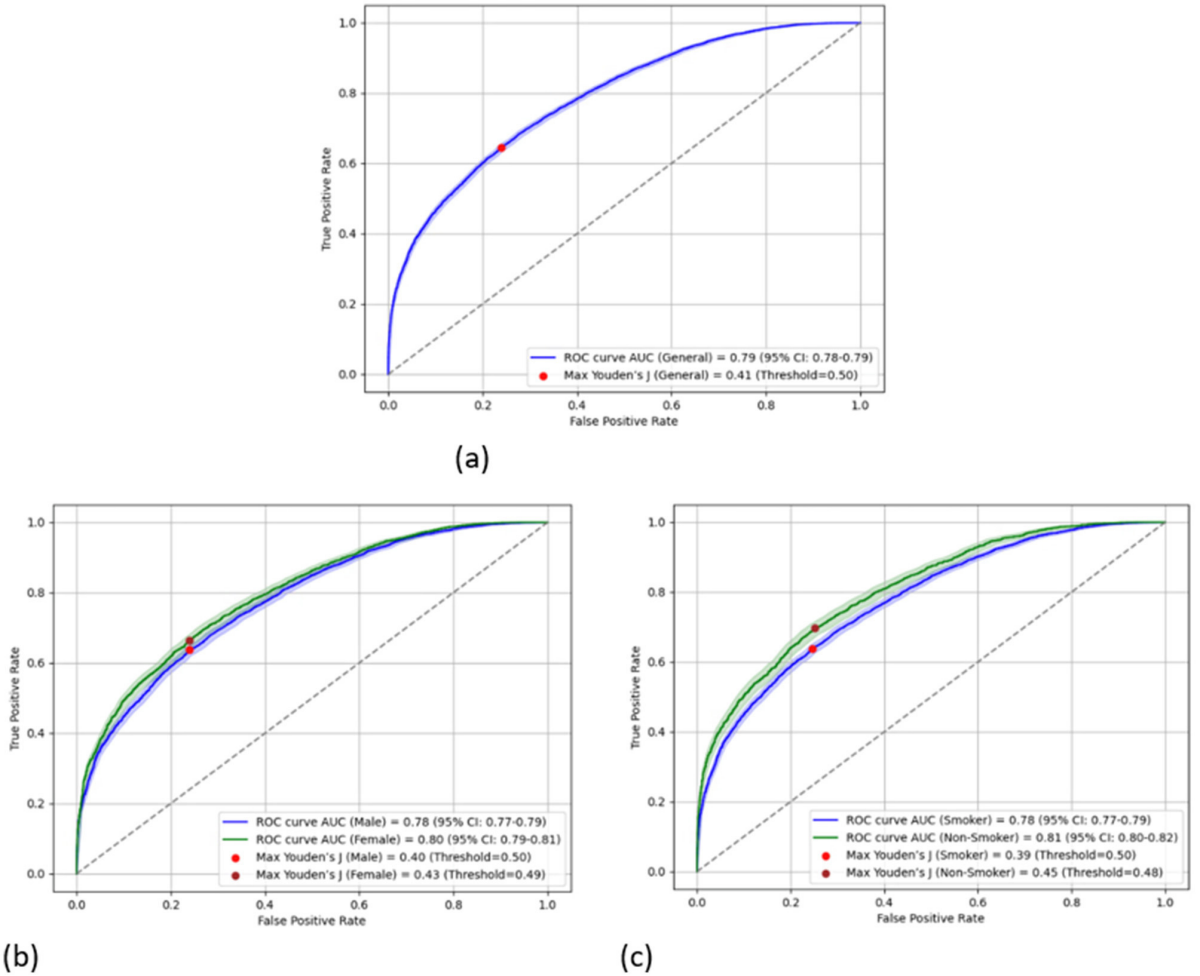
Calibration graph of the obtained model. The gray-dotted line indicates a perfect calibration. **(a)** The overall population. **(b)** Divided into genders. **(c)** Divided into smokers and non-smokers.

Figure 2 outlines a calibration plot (also called a reliability diagram), which illustrates how well the obtained model's predicted probabilities reflect actual outcomes. In Figure 2, the obtained calibration line is close to the diagonal line, which indicates that the obtained model is satisfactorily well-calibrated. Specifically, at low probabilities, the model slightly underestimates risk, while at higher probabilities, it slightly overestimates risk, which is a common artifact of machine learning models (37).

**Figure 3:**
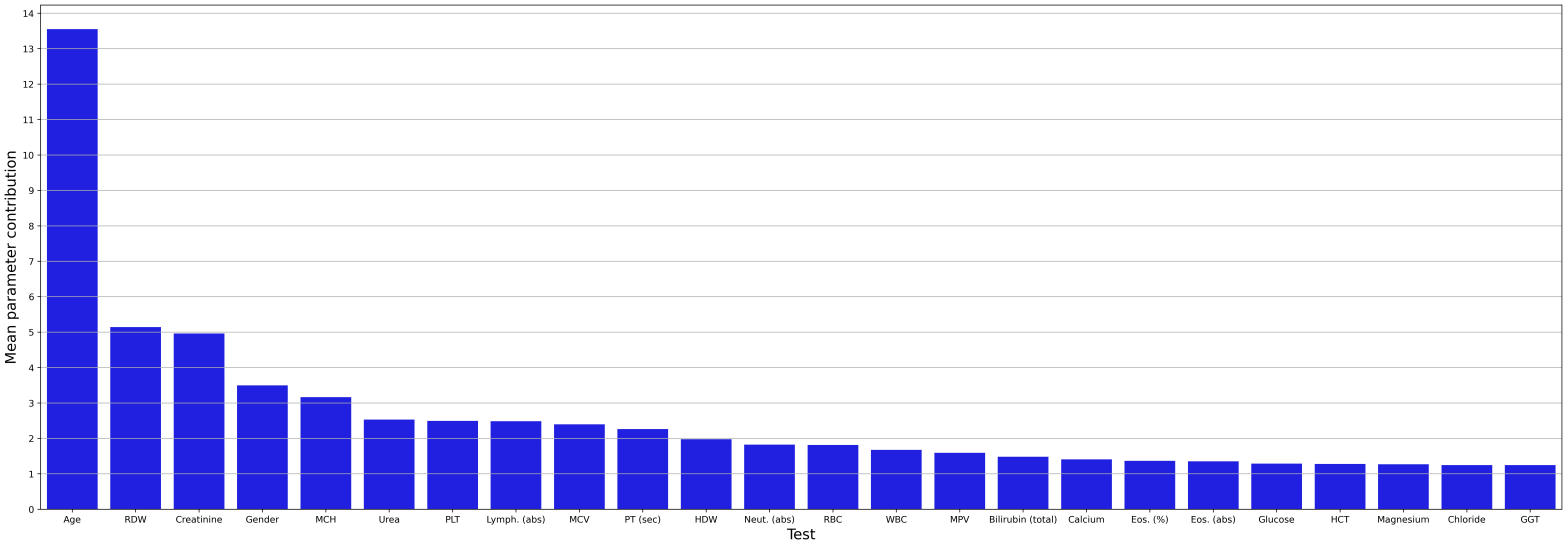
Mean contribution (importance) of each parameter to the AI model. abs, absolute; Eos, eosinophiles; GGT, gamma-glutamyl transferase; HCT, hematocrit; HDW, hemoglobin distribution width; Lymph, lymphocyte; MCV, mean corpuscular volume; MCH, mean corpuscular hemoglobin; MPV, mean platelet volume; Neut, neutrophil; PLT, platelets; PT, prothrombin time; RBC, red blood cells; RDW, red blood cell distribution width; sec; second; WBC, white blood cells.

Figure 3 shows the mean contribution (importance) of each parameter to the AI model. Apart from age and gender, we found 22 blood tests that significantly contribute to the AI model. Age was the most significant contributor (13.6%), followed by the red blood cell distribution width (RDW) (5.1%), creatinine (5%), gender (3.6%), and mean corpuscular hemoglobin (MCH) (3.3%). As the obtained model presents promising performance, we explored its decision-making process.

**Figure 4:**
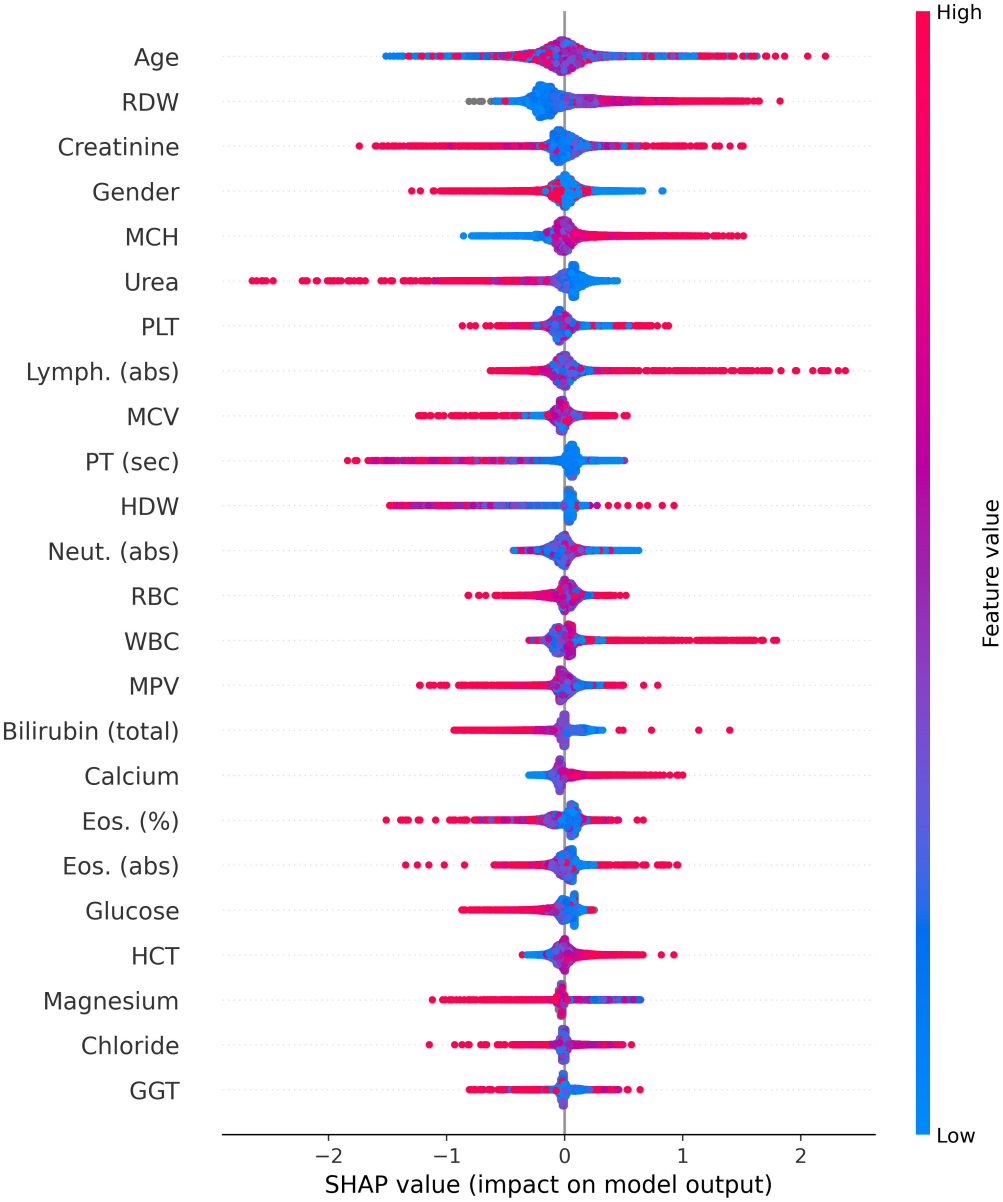
Shapley Additive Explanations (SHAP). abs, absolute; Eos, eosinophiles; GGT, gamma-glutamyl transferase; HCT, hematocrit; HDW, hemoglobin distribution width; Lymph, lymphocyte; MCV, mean corpuscular volume; MCH, mean corpuscular hemoglobin; MPV, mean platelet volume; Neut, neutrophil; PLT, platelets; PT, prothrombin time; RBC, red blood cells; RDW, red blood cell distribution width; sec; second; WBC, white blood cells.

Consequently, Figure 4 is a Shapley Additive Explanation (SHAP) in which each test result (parameter) is represented by its contribution to the model. For each parameter, the *x*-axis indicates its contribution to the LC risk, where values above 0 increase the risk and values below 0 reduce the risk linearly. The tests' results are organized with lower contribution importance to the model from top to bottom. For each test, the redder the color, the higher the value relative to the mean of that specific test result, and the bluer the color, the lower the value.

For gender, the red color represents women, and the blue color represents men. As mentioned above (Figure 3), age was identified as the most important parameter for the model. However, the SHAP distribution of this parameter reveals inconsistent behavior. As such, its effect in this age-matched group was not found to be strongly associated with LC, but rather diluted across the model. The RDW contributed the second most to the mode, with lower values from the mean indicating a possible relation to a lower risk of LC, while higher values potentially indicating an increased risk.

For creatinine, lower values were not found to be associated with LC risk; however, higher values did not contribute to the risk. Women were found to be associated with a lower risk of LC in contrast to men. For MCH and calcium, higher and lower values were found to be potentially related to increased and decreased LC risk, respectively. On the other hand, for urea and prothrombin time (PT) (seconds), higher and lower levels were found to be potentially associated with decreased and increased LC risk, respectively.

Higher white blood cell (WBC) counts were found to be potentially associated with an increased risk of LC, particularly concerning higher lymphocyte counts; however, lower levels of neutrophils may be associated with a reduced risk. Higher hematocrit (HCT) values were found to be related to an increased risk of LC, while higher levels of magnesium were found to be associated with a reduced risk. For the remaining blood tests, in the majority of cases, lower levels were not found to alter the risk for LC, while increased levels may signal either a higher or lower LC risk.

## Discussion

In this study, we evaluated the performance of a unique ML algorithm in predicting lung cancer (LC) and identifying the most prominent laboratory tests for forecasting LC. The overall accuracy for LC prediction was 71.2%, with an AUC of 78.7%, while the best accuracy was 73.6% among the never-smoker group, with an AUC of 78.4%. This finding could be partially explained by the matching in smoking backgrounds between the LC group and the control group. On the other hand, it is possible that the never-smoker group reflects biogenetic traits associated with cancer risk other than smoking-related effects on blood tests. Genetics and/or other environmental factors, other than smoking, could affect both blood test results and LC risk. These changes could be better demonstrated without smoking consequences. Among ~600 laboratory test analyses, we identified 24 main indices contributing to the predictive model.

In a study that compared different risk prediction models utilizing known risk factors for LC, the PLCOm2012 model (which uses age, race, education, body mass index, COPD, personal history of cancer, family history of lung cancer, smoking status, smoking duration, smoking intensity, and years since cessation) was found to best predict LC risk with an AUC of 77%, a sensitivity of 83%, and a specificity of 62.5% (38). In our model, the AUC was slightly higher than that of the PLCOm2012 model (78.7%) and higher than that of the National Lung Screening Trial (NLST) inclusion criteria model, in which the AUC was 68%. However, in our study, the sensitivity was lower (63%), and the specificity was higher than that of the PLCOm2012 and the NLST models (77.2 vs. 62.5% and 62.2%, respectively) (39). Since, to the best of our knowledge, there are no existing models that use multiple blood tests for LC prediction, our study is a pioneering effort to explore this field. Further studies need to be performed in larger and more diverse populations before incorporating blood tests into other traditional models and expanding them to include non-smokers and female populations.

Age, as expected, was identified as the most important parameter of the model. However, the SHAP distribution of this parameter reveals inconsistent behavior. Consequently, in this matched group, the effect of age (along with smoking behavior and gender) was found to be rather diluted across the model.

It is important to emphasize that, when we talk about lower or higher levels, we are not referring to values below or above normal ranges, but rather to values that are relatively lower or higher compared to the average of the study population. Moreover, by definition, AI models are very complex as they capture high-dimensional, non-linear correlations between all parameters. For some patients, these correlations indicate that high levels of one parameter could increase the risk of LC, given the results of the other parameter. In contrast, for other patients, lower levels of a different parameter could increase the risk. For this reason, some parameters in the proposed model appear to behave chaotically, with both higher and lower levels potentially increasing or decreasing the risk.

### Hematologic parameters

The RDW made the second-highest contribution to the model, with values lower than the mean being potentially associated with a lower risk of LC and higher values indicating an increased risk. A growing body of research supports RDW as a potential biomarker for both the diagnosis and prognosis of various malignancies (40, 41). Song et al. (40) reported a significant increase in RDW among NSCLC patients, enabling discrimination of NSCLC patients from healthy participants with a specificity of 76% and a sensitivity of 76%. A potential explanation is that high RDW has been demonstrated to be an independent risk factor for poorer prognosis in non-elderly patients with esophageal cancer, but not in elderly patients with esophageal cancer, in whom other comorbidities could lead to elevated RDW values (42). Nevertheless, it is noteworthy that, after adjusting for other hematological and inflammatory factors, RDW frequently ceased to exhibit a significant association with cancer risk and mortality (41), suggesting that the association between RDW and cancer primarily reflects the involvement of RDW in inflammation and oxidative stress, which are, in fact, risk factors for cancer (41, 43).

Other red blood cell parameters that have been shown to influence LC prediction in this study are MCH and HCT. For MCH, higher values may be associated with an increased risk of LC, and lower values may be associated with a lower risk. Zhang et al. (44) found that MCH levels before treatment could serve as a predictive marker linked to disease-free survival (DFS) in breast cancer, as patients in the higher MCH group exhibited shorter DFS times than those in the lower MCH group. In another study, in patients with hepatocellular carcinoma (HCC), MCH was independently associated with overall survival (OS) and, as such, may be valuable in evaluating the prognosis of patients who undergo hepatectomy (45).

In this study, elevated levels of HCT were found to be associated with an increased risk of LC. A possible explanation could be the high prevalence of smoking among LC patients, which may raise HCT levels.

In this study, lower PT in seconds was found to be potentially associated with an increased risk of LC, while higher levels were associated with a lower risk. Previous studies demonstrated that ~50% of cancer patients and up to 90% of those with metastatic disease have abnormal clotting tests. These tests may show a mild extension or shortening of PT or activated partial thromboplastin time, and increased levels of fibrinogen (46). Therefore, the higher risk of LC may be explained by a hypercoagulable state associated with lower PT levels.

In this study, elevated WBC counts with higher absolute lymphocytes and, in some patients, lower absolute neutrophil counts were found to be potentially associated with an increased risk of LC. Reduction in neutrophil counts was also found in another study as a marker for LC diagnosis (47). On the other hand, according to Sahin the neutrophil/lymphocyte ratio (NLR) is higher in LC patients, aiding in the diagnosis and detection of late-stage disease (48).

### Biochemistry parameters

In this study, high serum calcium levels were associated with a higher LC risk, while lower levels were associated with a lower risk. A possible explanation for this observation is that elevated calcium values may indicate advanced disease with bone metastases or paraneoplastic syndrome. Thus, since the stage of the disease was unknown in this study, we could not verify this observation. It is plausible that patients with low calcium levels may be cancer-free. Moreover, according to an observational study published in 2021, calcium concentration is an independent risk factor for brain metastasis prediction in NSCLC patients (49).

Higher creatinine levels were found to be potentially associated with either lower or higher LC risk, while lower levels were found to either increase or decrease the risk. To the best of our knowledge, there is currently no association between low creatinine levels and LC incidence. On the other hand, an impressive dichotomous distribution was found for urea (fifth in model importance), in which higher urea levels were associated with decreased LC risk, and had the highest impact (long left arm) among all the other parameters. In addition, lower levels slightly but consistently reduced the degree of risk. This interesting finding aligns with a recent large study that reported a lower incidence and mortality rate among patients with high serum urea levels for several solid cancers, especially stomach, esophageal, and LC (50). The authors of that study suggested that excessive urea can cause differences in DNA damage (e.g., breakage of chromosome 3p but not chromosome 3q), gene regulation, and alterations in urea cycle metabolites. Conversely, in a small study by Chang et al., a model was proposed for LC risk assessment in which the higher the blood urea nitrogen (BUN) levels, the greater the risk of LC (51). Further studies are needed to explore this highly complex field.

The findings of our study have several limitations. Although, as a retrospective study, there was a massive amount of information from the EMR, significant heterogeneity in the data collected in the past and its timing influenced the ML results. Since this study only analyzed blood tests with respect to age, gender, and smoking habits, other confounding factors, such as comorbidities, drug use, and socioeconomic factors, were not measured. In addition, data concerning smoking were incomplete. Information on smoking quantity was lacking for many patients, was not dated in all cases relative to the LC diagnosis, and was not collected frequently enough for the entire study population. In addition, the lack of data on tumor histologic subtypes and stages is another shortcoming of our research. Furthermore, since we used an ML-based modeling approach in this study, the explainability of the CatBoost model was limited and did not provide much clinical insight. Finally, the study's cohort was only based on Israeli data, which may introduce cultural, ethnic, environmental, and clinical practices biases and could not be fully generalized. Future research may address these limitations by collecting data from multiple international centers and using causality models. Furthermore, integrating blood test analysis with other EMR components, such as AI LDCT analysis, comorbidities, drug use, and demographic parameters, could increase model accuracy and sensitivity in future studies.

In conclusion, the proposed AI model demonstrates good predictive capability, achieving 71.2% accuracy in the validation cohort for LC using only blood test results. Notably, the model demonstrated better accuracy (73.6%) for never smokers compared to smokers (70.1%). In addition, basic science studies were conducted to shed light on and provide explanations regarding the connections between the specific blood results and LC. Further studies should be conducted to validate these results.

## Data Availability

According to Israeli law, the data is not publicly available. Requests to access the datasets should be directed to dekels1@zahav.net.il.
