# Correction: A blood test-based machine learning model for predicting lung cancer risk

**DOI:** 10.3389/fmed.2025.1654707

**Published:** 2025-07-11

**Authors:** Lihi Schwartz, Naor Matania, Matanel Levi, Teddy Lazebnik, Shiri Kushnir, Noga Yosef, Assaf Hoogi, Dekel Shlomi

**Affiliations:** ^1^Fliner Clinic, Department of Family Medicine, Dan-Petah-Tiqwa District, Clalit Health Services Community Division, Petah Tiqwa, Israel; ^2^Department of Computer Science, Bar Ilan University, Ramat Gan, Israel; ^3^Adelson School of Medicine, Ariel University, Ariel, Israel; ^4^Department of Mathematics, Ariel University, Ariel, Israel; ^5^Department of Cancer Biology, Cancer Institute, University College London, London, United Kingdom; ^6^Research Authority, Rabin Medical Center, Beilinson Campus, Petah Tiqwa, Israel; ^7^Research Unit, Dan-Petah-Tiqwa District, Clalit Health Services Community Division, Ramat Gan, Israel; ^8^The School of Computer Science and The Data Science and Artificial Intelligence Research Center, Ariel University, Ariel, Israel; ^9^Pulmonary Clinic, Dan-Petah-Tiqwa District, Clalit Health Services Community Division, Ramat Gan, Israel

**Keywords:** lung cancer, artificial intelligence, machine learning, blood test, prediction model

The order of authors in the author list of the published paper was erroneously given as:

Lihi Schwartz^1*†^, Naor Matania^2†^, Matanel Levi^3^, Shiri Kushnir^4^, Noga Yosef^5^, Assaf Hoogi^6^, Teddy Lazebnik^7, 8‡^ and Dekel Shlomi^3, 9‡^.

The correct author list reads:

Lihi Schwartz^1*†^, Naor Matania^2†^, Matanel Levi^3^, Teddy Lazebnik^4, 5^, Shiri Kushnir^6^, Noga Yosef^7^, Assaf Hoogi^8^, Dekel Shlomi^3, 9^.

Affiliations were reordered and extended for Shiri Kushnir and Assaf Hoogi. The correct affiliation list is below:

^1^Fliner Clinic, Department of Family Medicine, Dan-Petah-Tiqwa District, Clalit Health Services Community Division, Petah Tiqwa, Israel.

^2^Department of Computer Science, Bar Ilan University, Ramat Gan, Israel.

^3^Adelson School of Medicine, Ariel University, Ariel, Israel.

^4^Department of Mathematics, Ariel University, Ariel, Israel.

^5^Department of Cancer Biology, Cancer Institute, University College London, London, United Kingdom.

^6^Research Authority, Rabin Medical Center, Beilinson Campus, Petah Tiqwa, Israel.

^7^Research Unit, Dan-Petah-Tiqwa District, Clalit Health Services Community Division, Ramat Gan, Israel.

^8^The School of Computer Science and The Data Science and Artificial Intelligence Research Center, Ariel University, Ariel, Israel.

^9^Pulmonary Clinic, Dan-Petah-Tiqwa District, Clalit Health Services Community Division, Ramat Gan, Israel.

The author contributions statement was erroneously given as LS: Investigation, Writing – original draft. NM: Formal analysis, Investigation, Methodology, Software, Validation, Visualization, Writing – review & editing. ML: Investigation, Formal analysis, Methodology, Software, Visualization, Writing – review & editing. SK: Data curation, Writing – review & editing. NY: Project administration, Writing – review & editing. AH: Conceptualization, Funding acquisition, Writing – review & editing. TL: Conceptualization, Formal analysis, Investigation, Methodology, Resources, Software, Supervision, Validation, Visualization, Writing – original draft, Writing – review & editing. DS: Conceptualization, Investigation, Supervision, Writing – original draft, Writing – review & editing.

The correct author contributions statement is

LS: Investigation, Writing – original draft. NM: Methodology, Software, Formal analysis, Investigation, Validation, Visualization, Writing – review & editing. ML: Investigation, Software, Formal analysis, Writing – review & editing. TL: Conceptualization, Methodology, Software, Formal analysis, Investigation, Validation, Visualization, Supervision, Writing – original draft, Writing – review & editing. SK: Data curation, Writing – review & editing. NY: Project administration, Writing – review & editing. AH: Conceptualization, Methodology, Funding acquisition, Writing – review & editing. DS: Conceptualization, Methodology, Investigation, Supervision, Writing – original draft, Writing – review & editing.

The statement “^‡^These authors have contributed equally to this work and share last authorship” has been removed. Author Teddy Lazebnik was erroneously assigned as equally contributing to the last author and erroneously sharing last authorship.

The original version of this article has been updated.

